# Merkel Cell Carcinoma of the External Ear: Population-Based Analysis and Survival Outcomes

**DOI:** 10.3390/cancers14225653

**Published:** 2022-11-17

**Authors:** André S. Alves, Matteo Scampa, Jérôme Martineau, Salvatore Giordano, Daniel F. Kalbermatten, Carlo M. Oranges

**Affiliations:** 1 Department of Plastic, Reconstructive, and Aesthetic Surgery, Geneva University Hospitals, Geneva University, 1205 Geneva, Switzerland; 2 Department of General and Plastic Surgery, Turku University Hospital, University of Turku, 20521 Turku, Finland

**Keywords:** Merkel Cell Carcinoma, external ear, skin, UV, SEER, survival analysis, epidemiology, malignant

## Abstract

**Simple Summary:**

Merkel Cell Carcinoma (MCC) is a neuroendocrine skin tumor with malignant characteristics. Multiple regions can be affected, and tumoral incidence and outcomes vary depending on the primary site. A multidisciplinary team management is of paramount importance to offer the best treatment options to patients. Due to the low incidence of MCC, fine analysis of specific regions is limited, particularly the external ear. We performed a population-based analysis of demographic and survival outcomes of external ear MCC. The aim of this study is to offer updated data on tumor behavior and survival outcomes of this specific region. The male gender, an age over 80 years old and a tumor size above 5 cm were risk factors for low overall survival. Gross (<1 cm) and wide (>1 cm) margins of surgical excision offer the best overall survival.

**Abstract:**

(1) **Background**: Due to its highly aggressive behavior, the ability to identify and manage Merkel Cell Carcinoma (MCC) with a full understanding of its characteristics is essential. Because the external ear is an exposed area, resection can have dramatic consequences on patient’s self-image, which is why it is fundamental to detect MCC, typically found on UV-exposed regions such as the ears, at an early stage. (2) **Methods**: The Surveillance, Epidemiology, and End Results (SEER) database was searched for all external ear MCC between 2000 and 2019. A descriptive analysis based on frequencies was made to describe the demography of pathophysiologic features linked to MCC. Overall survival (OS) was studied and compared between variables with a log rank test. A multivariable Cox regression analysis was then computed to identify independent prognostic factors. (3) **Results**: A total of 210 patients (160 men) were identified with a median age of 80 years. The median OS was 47 months. Factors associated with lower OS included an age of over 80 years, the male gender, a tumor size of >5 cm, and metastatic disease. Gross (<1 cm) and wide (>1 cm) surgery excision margins were the surgery types with the best OS. (4) **Conclusions**: MCC of the external ear is diagnosed mostly in old men. Among the 182 patients who received a surgical procedure, gross and wide excision without radiotherapy were associated with the best OS.

## 1. Introduction

Merkel Cell Carcinoma (MCC) is a rare but malignant skin tumor with an incidence rate of about 0.7 cases per 100,000 persons-year in the United States [1]. This pathology has a neuroendocrine component and is mainly associated with chronic UV exposure and polyomavirus infection [2,3]. Immunosuppressed individuals have been identified to be at risk for MCC. Because of low skin UV phototype and decreased immunity, old white men are more likely to develop this skin tumor [4]. The head and face are the most affected regions because of their inherent higher exposure to UV [5,6]. Cases of lower limb MCC have also been reported and are often associated with a Merkel cell polyomavirus (MCPyV) infection and immunosuppression, and they have better overall survival compared to other localizations of MCC [7,8]. MCC is typically described as a solitary cutaneous or subcutaneous nodule [9,10].

Current treatment guidelines rely on initial staging, based on the TNM classification. In most case, surgical excision with margins is the recommended treatment. In the case of advanced disease, radiotherapy, chemotherapy, and immunotherapy are commonly used [11,12]. A close multidisciplinary team approach, in which plastic surgeons are involved for the resection and reconstruction, is required, as the surgical treatment of MCC of the head and neck region can be challenging when wide surgical excision is needed, while trying to preserve aesthetic subunits. The resection of the external ear MCC is particularly difficult from a surgical standpoint, with specific anatomical feature comprehension being crucial in the reconstruction planning, due to the paucity of soft tissues and sequelae that can potentially lead to self-image distortion [13]. To help improve overall and surgical outcomes, a precise knowledge of tumor behavior and treatment patterns is required.

Epidemiologic studies have already been published on large datasets but were mostly based on global head and neck localization with no studies focusing specifically on the external ear [8,14,15,16]. This UV-exposed region may go unnoticed in older people and the best therapeutic strategy is yet to be defined. The aim of this study is to present a population-based analysis of survival and surgical outcomes of the external ear to improve patient care.

## 2. Materials and Methods

### 2.1. Patient Selection

All cases of external ear MCC between 2000 and 2019 were retrieved from 17 different registries of the Surveillance, Epidemiology and End Results (SEER) program. This database regroups 17 US cancer registries under the supervision of the National Cancer Institute. SEER*stat software 8.4.0.1 (seer.cancer.gov/seerstat, U.S.) was used to extract all variables using the case listing option. Patients were identified using the International Classification of Disease for Oncology, third edition (ICOD-3) codes: 8247/3 for MCC and C44.2 for external ear as tumor primary site.

### 2.2. Variable Selection

A wide range of features were selected to highlight the actual epidemiologic state of MCC on the external ear. Demographic variables such as sex, race, age at diagnosis and living area were identified to obtain an overview of the affected population group. Age was subdivided in three categories: <65, 65–79, ≥80. These three categories of ages were chosen because MCC is a tumor affecting the elderly with a median age at diagnosis above 75 years. For this reason, we tried to subdivide patients into a fitter younger group (<65) and a vulnerable age group (≥80) who are expected to have a lower survival rate. Regarding the disease characteristics, TNM classification, SEER stage, the presence of other tumors, the type of treatment, and survival data were extracted to obtain an overview of this pathology and the management care process (Table 1).

The SEER*stat merging tool was used to create a variable combining the type of surgical resection in 3 categories with the use of radiotherapy: local excision without margin, gross excision with <1 cm margin, and wide excision > 1 cm margin. A merged variable for multimodal treatment, chemotherapy with radiotherapy and chemotherapy without radiotherapy, was also created (Table 2 and Table 3). Unfortunately, because it was not reported in the database, we could not include immunotherapy in the analysis.

A variable for UV exposure and a variable based on median household income were created. For the first variable, the number of sunny days during the year in each US state was retrieved from the National Weather Service 2017 UV index report. As performed by Scampa et al, we summed the days with UV index defined as “extreme”, “very high”, and “high”. US states with ≥180 days of sun exposure were classified as high UV exposure and US states with <180 days as low UV exposure. In the first group, we included Hawaii (308 days), Louisiana (222 days), California (219 days), New Mexico (213 days), and Georgia (180 days), while Utah (166 days) Kentucky (141 days), New Jersey (136 days), Iowa (125 days), Connecticut (115 days), and Seattle (101 days) were included in the low UV exposure group [8].

Data on median household income in each US state were retrieved from the US Census Bureau. US states were separated into two groups; the first group included the 25 US states with the highest median household income (New Jersey: 85,751, Hawaii: 83,102, California: 80,440, Connecticut: 78,833, Seattle: 78,687, Utah: 75,780) and the second group included the 25 states with the lowest income (Georgia: 61,980, Iowa: 61,691, Kentucky: 52,295, New Mexico: 51,945, Louisiana: 51,073).

### 2.3. Statistical Analysis

Data were processed using IBM SPSS version 28 (I.B.M., Armonk, NY, USA). Demographic and tumor characteristic were described. Unknown values were not included in the percentual calculation.

Overall survival (OS) was calculated using the Kaplan–Meier method. Univariable analysis of sex, age, race, UV exposure, household income, TNM, and treatment was compared using a log-rank test.

A multivariable Cox regression was used to identify independent prognostic factors for age, sex race, tumor stage and treatment. Each variable was individually adjusted for age and sex as they were previously identified to have an influence on OS. A *p*-value under 0.05 was considered statistically significant.

## 3. Results

**Figure 1:**
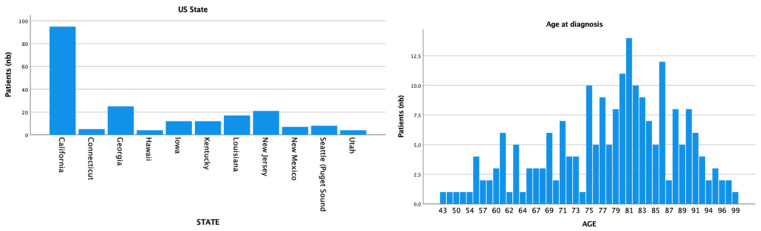
Registry location and age at diagnosis distribution.

A total of 210 patients with external ear MCC were retrieved. The mean age of our population was 78 (SD = 10.8) and the median age was 80, ranging from 43 to 99 years. Patients were mostly white (*n* = 191; 91%), of male gender (*n* = 160; 76.2%), and from California (*n* = 95; 45.2%) (Figure 1). The majority of patients identified were from US states with a high median household income (*n* = 129; 63.8%) and high UV exposure (*n* = 148; 70.5%). For the tumor staging, T1 (*n* = 64; 68.1%) with N0 (*n* = 92; 65.2%) and M0 (*n* = 131; 88.5%) were the most common classifications.

**Figure 2:**
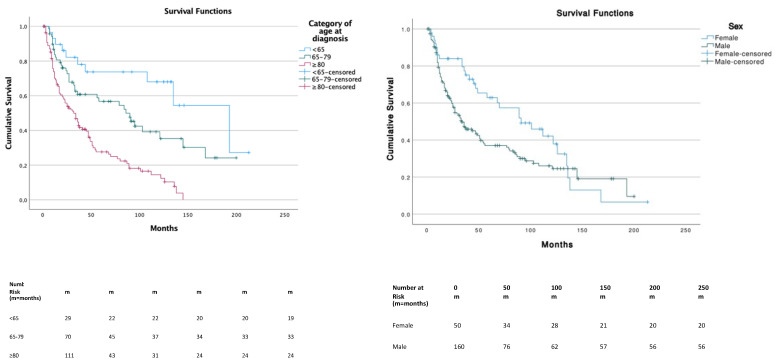
Survival distribution according to age and gender.

The median OS was 47 months (95% CI, 34.7–59.2) and significantly differed across the three age categories. Patients in the ≥80 years age group had a significantly lower survival compared to other age groups (*p* < 0.01) with a median OS (mOS) of 32 months (95% CI, 20.6–43.4). Patients in the <65 years age group had a better overall survival compared to ≥80 years (*p* = 0.000) and 65–79 years (*p* = 0.041), with a mOS of 193 months (95% CI, 109.1–276.9). Patients in the 65–79 years age group had a mOS of 86 months (95% CI, 51.8–120.2). Survival between sex was significantly (*p* = 0.038) lower in males (median, 34 months; 95% CI, 21.8–46.1) compared to females (median, 91 months; 95% CI, 48.8–133.2) (Figure 2). Among races, no significative differences were underlined.

**Figure 3:**
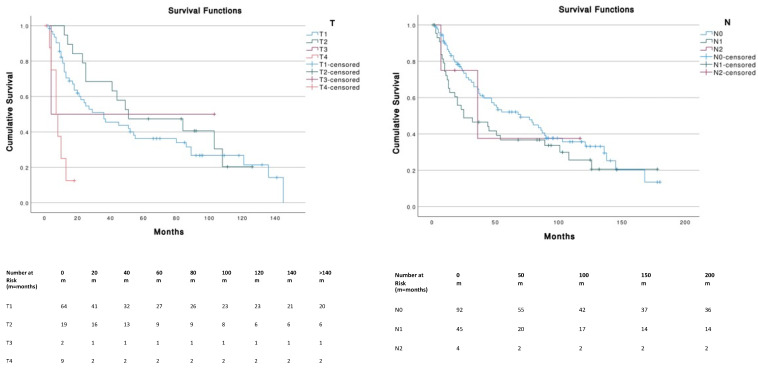

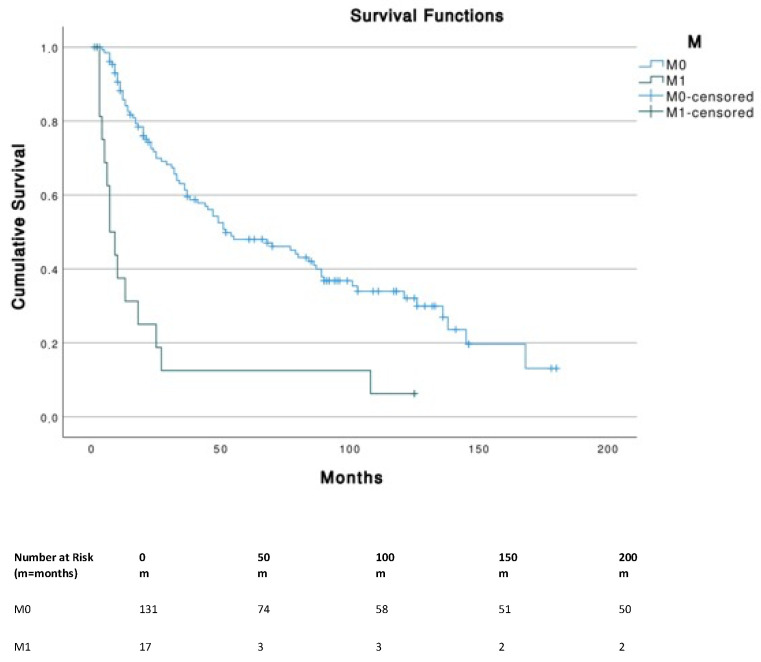
Survival curves according to TNM stages.

Patients with tumor sizes ≤ 5 cm corresponding to T1 (≤2 cm) and T2 (2–5 cm) had a significantly (*p* < 0.001) higher OS compared to T4 (i.e., primary tumor invades fascia, muscle, cartilage or bone). No significant differences were found across different N stages. The presence of metastasis (M1) was associated with a significantly (*p* < 0.001) lower survival (median, 7 months; 95% CI, 3.1–10.9) compared to M0 (median, 52 months; 95% CI, 26.3–77.7) (Figure 3).

**Figure 4:**
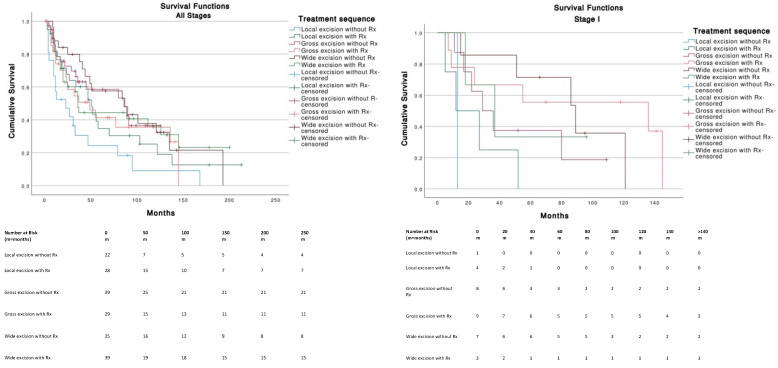

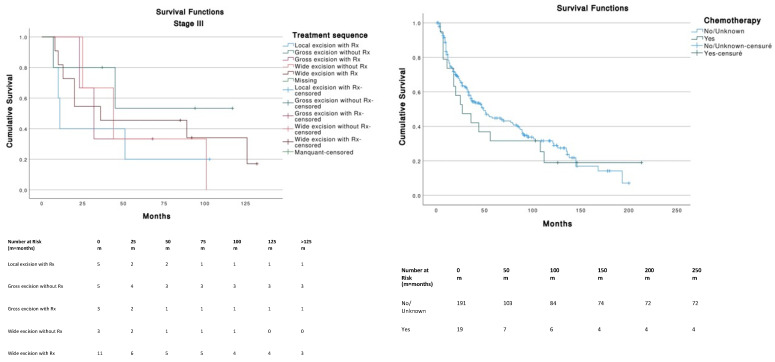
Tumor staging and therapeutic strategies survival curves.

For Stage I tumors (T1N0M0), wide excision without radiotherapy (Rx) was the best therapeutic strategy among other treatments and was significantly better than local excision with (*p* = 0.013) or without Rx (*p* = 0.008). Gross excision with Rx was significantly better (*p* = 0.047) compared to local excision with Rx (Figure 4). For Stage III tumors (T0-T4, N1-3, M0), no significant differences in survival were found across treatments. Stages II (*n* = 11) and IV (*n* = 13) were not analyzed because of insufficient data. The use of chemotherapy (median, 27 months; 95% CI, 4.2–49.7) or the absence of chemotherapy (median, 49 months; 95% CI, 34.2–63.7) was not significantly (*p* = 0.586) associated with a better survival.

Age at diagnosis, sex, race, TNM, stage and therapeutic strategy were identified as independent prognostic factors in the multivariate Cox regression analysis (Table 4). Gross (<1 cm) and wide (>1 cm) margins excision without radiotherapy were the therapeutic strategies that offered the best overall survival with a median OS of 87 months (95% CI, 32.3–141.7) and 86 months (95% CI, 31.4–140.5), respectively. Local excision without radiotherapy was the treatment associated with the lowest overall survival, with a median OS of 23 months (95% CI, 1.8–44.2).

## 4. Discussion

To our knowledge, this is the first and largest population-based study on survival outcomes focusing specifically on MCC of the external ear. Similarly to other body localizations, our results show that MCC preferentially affected white elderly men [17].

UV exposure has been associated with an increased risk of developing a skin tumor such as MCC [6,18]. The worldwide distribution of this tumor varies significantly across countries and supports UV association with MCC physiopathology. Nordic countries, such as Sweden, have a lower MCC incidence (0.3 cases per 100,000 persons–year) compared to southern places such as Queensland in Australia (1.6 cases per 100,000 persons–year) where sun exposure is higher [19,20]. The pigmentary phototype is an important predisposing factor for risk exposure to light [21]. Some specific genes have also been linked to enhanced DNA damage with chronic UV exposure [3].

Compared to other MCC localizations, the affected patients were older at diagnosis [8]. The OS was worse for patients over 80 years old. Immunodeficiency appeared to be the main explanation given the higher susceptibility of DNA damage and mismatch repair default as age increases [22]. The prevalence to encounter more than one tumor and metastatic disease is also more frequent with age and predisposes to a lower survival rate.

The male gender was linked to a significantly worse OS in our study—in line with the study of Tam et al. which showed that women had a survival advantage in MCC. They concluded that the reason for better survival in women was unclear, although they hypothesize it may be related to stronger immune responses in women [23].

Tumor size according to the TNM classification was significantly associated with a worse OS in patients presenting a T4 tumor at diagnosis. This is in accord with the study of Lamberti et al. in which the authors postulate that the worse OS in patients presented with T4 tumors is likely due to an occult systemic disease, and the effect of local treatments is, thus, diminished [24]. In our study, OS was not significantly different across groups based solely on the lymph node status, which can probably be explained by the limited sample size. Indeed, a positive lymph node status in MCC was shown to be associated with worse outcomes [25,26]. Unsurprisingly, metastatic disease was associated with a lower survival rate. The effect of the number of tumors was analyzed but had no significant influence on OS. Overall survival varied significantly depending on the TNM stage at diagnosis—it was significantly better in Stage I cases and significantly worse in Stage IV cases.

Primary site surgery has become the main standing therapeutic strategy for Merkel Cell Carcinoma. Clear evidence shows that adequate surgical margins are essential to prevent recurrence and a negative margin status is associated with a better survival rate [27]. As demonstrated in this study, wide excision was the best therapeutic approach, consistent with the results of previous studies [28,29].

Following tumor excision, external ear reconstruction is important to restore function and a proper esthetic appearance. Multiple techniques are used including skin grafts, local flaps, rotation flaps, pedicled flaps, pre-auricular pull-through flaps, and even prosthetic reconstruction in cases of auriculectomy [30,31]. Proper pre-operative planning is needed to ensure a satisfactory reconstruction.

Radiation treatment as an adjuvant therapy has also been associated with lower recurrences and improved local control [32,33,34,35]. Conversely, some studies did not find a significant improvement with survival and outcomes [36,37,38,39]. In our study, the use of radiation therapy did not yield better overall survival outcomes. One explanation could be that radiation was preferentially used for aggressive MCC, already associated with a worse prognosis. However, in our study, even in patients with same tumor stages, the use of radiotherapy was not found to be significantly beneficial for OS. This is important to consider, given that preoperative radiotherapy is linked to higher morbidity and can sometimes lead to temporal bone osteoradionecrosis—a complication that often requires treatment with regional flaps or free tissue transfers [40,41]. In this study, the use of chemotherapy was, similarly, not associated with a better survival in patients that received this treatment on the whole, when compared to MCC affecting other locations, we found that external ear MCC was more frequent in older males and that there was no added benefit from radiotherapy. We noted no other specific features associated with this location that should be taken into account in routine clinical practice.

As with most multi-centric retrospective studies, the current population-based study was assessed based on data collection and patient records that could lead to some bias. Other limitations also exist regarding the reported data from the SEER program. For instance, summary stage is only reported since 2004. Missing data, such as immunotherapy, radiation doses, and patient’s comorbidity, limit the observation and the evidence of our study in relation to current therapeutic guidelines. Due to the non-binomial value of the treatments analyzed in this study, a propensity score-matched approach was not deemed possible. Therefore, treatment outcomes might be influenced by confounding factors, as treatment choice is highly dependent on the characteristics of both patients and tumors.

## 5. Conclusions

This study provides comprehensive knowledge on Merkel Cell Carcinoma of the external ear. To our knowledge, it is the first study analyzing survival outcomes in MCC of the external ear. It offers tools to better understand the demographic features of the affected population and could help practitioners in counseling patients with regard to prognostic factors. Our data suggest that a surgical procedure with a wide excision margin of >1 cm provides the best overall survival. Involving plastic surgeons early in the management process is required, as preoperative planning of the resection surgery and the choice of the reconstructive technique are important to allow for optimal external ear reconstruction.

## Figures and Tables

**Table 1:** Demographic and pathological characteristics of the study population.

Variable	MCC on External Ear (*n* = 210)
Sex	
Female	50 (23.8%)
Male	160 (76.2%)
Age at diagnosis	
Mean (SD)	77.98 (10.785)
Median (min–max)	80.00 (43–99)
Race	
White	191 (91.8%)
Asian/Pacific Islander	10 (4.8%)
Black	6 (2.9%)
American Indian/Alaska Native	1 (0.5%)
State	
California	95 (45.2%)
Connecticut	5 (2.4%)
Georgia	25 (11.9%)
Hawaii	4 (1.9%)
Iowa	12 (5.7%)
Kentucky	12 (5.7%)
Louisiana	17 (8.1%)
New Jersey	21 (10%)
New Mexico	7 (3.3%)
Seattle	8 (3.8%)
Utah	4 (1.9%)
UV exposure	
≥180 days	148 (70.5%)
<180 days	62 (29.5%)
TNM	
T	
T1	64 (68.1%)
T2	19 (20.2%)
T3	2 (2.1%)
T4	9 (9.6%)
N	
N0	92 (65.2%)
N1	45 (31.9%)
N2	4 (2.8%)
M	
M0	131 (88.5%)
M1	17 (11.5%)
Stage	
Stage I	33 (36.3%)
Stage II	11 (12.1%)
Stage III (nodal)	32 (35.2%)
Stage IV (distant metastasis)	15 (16.5%)

**Table 2:** Treatment characteristics of the study population.

Variable	MCC on External Ear (*n* = 210)
Surgery	
No	28 (13.3%)
Local excision (no margin)	50 (23.8%)
Gross excision (<1 cm margin)	68 (32.4%)
Wide excision (>1 cm)	64 (30.5%)
Surgery/Radiotherapy (Rx)	
Local excision with Rx	28 (25.0%)
Local excision without Rx	22 (18.2%)
Gross excision with Rx	29 (37.9%)
Gross excision without Rx	39 (53.8%)
Wide excision with Rx	39 (38.5%)
Wide excision without Rx	25 (32.0%)
Multimodal treatment	
Surgery without Radiotherapy	86 (41.0%)
Surgery with Radiotherapy	96 (45.7%)
Radiotherapy without Chemotherapy	93 (44.3%)
Radiotherapy with Chemotherapy	16 (7.6%)
Radiotherapy	
Yes	109 (51.9%)
No/unknown	101 (48.1%)
Chemotherapy	
Yes	19 (9.0%)
No/unknown	191 (91.0%)

**Table 3:** Summary of the treatment sequences.

	No Radiotherapy and No Chemotherapy (*n* = 84)	Radiotherapy and No Chemotherapy (*n* = 83)	Chemotherapy and No Radiotherapy (*n* = 3)	Radiotherapy and Chemotherapy (*n* = 16)
Local Excision (*n* = 50)	22	24	0	4
Gross Excision (*n* = 68)	38	27	1	2
Wide Excision (*n* = 64)	24	32	1	7
No Surgery (*n* = 28)	0	0	1	3

**Table 4:** Multivariate Cox regression analysis.

Variable	B	*p* Value	Exp (B)	95% CI for Exp (B)
Age ^a^				
<65				
65–79	−1.626	<0.001	0.197	0.100/0.386
≥80	−0.897	<0.001	0.408	0.271/0.613
Sex ^a^				
Male				
Female	−0.624	0.003	0.536	0.353/0.813
Tumor Size ^a^				
T1		0.000		
T2	−0.173	0.589	0.841	0.450/1.575
T3	−0.823	0.422	0.439	0.059/3.274
T4	1.769	0.000	5.864	2.437/14.106
Lymph node status ^a^				
N0		0.449		
N1	0.227	0.217	1.323	0.848/2.064
N2	0.723	0.686	1.339	0.324/5.529
Metastasis ^a^				
M0				
M1	1.712	0.000	5.539	3.023/10.149
Stage ^a^				
I		0.000		
II	0.036	0.936	1.036	0.433/2.482
III	−0.095	0.750	0.909	0.505/1.636
IV	1.447	0.000	4.248	2.082/8.667
Treatment ^a^				
Local excision without Rx		0.201		
Local excision with Rx	−0.575	0.079	0.563	0.297/1.068
Gross excision without Rx	−0.787	0.020	0.455	0.235/0.883
Gross excision with Rx	−0.744	0.029	0.475	0.244/0.928
Wide excision without Rx	−0.649	0.060	0.523	0.265/1.029
Wide excision with Rx	−0.642	0.042	0.526	0.283/0.978
Radiotherapy ^a^				
No/unknown				
Yes	−0.112	0.525	0.894	0.633/1.263
Chemotherapy ^a^				
No/unknown				
Yes	0.167	0.547	1.181	0.687/2.032

^a^ Individual variables were individually combined with age and sex for adjustment.

## Data Availability

SEER*stat software 8.4.0.1.
